# Molecular Evolution of Cu Transporters and Transcription Factors in Plant Response to Copper Stress

**DOI:** 10.3390/plants14172710

**Published:** 2025-09-01

**Authors:** Haiyang Tang, Qianqian Tang, Jin Zhang, Xuan Chen, Tao Tong, Qingfeng Zheng, Li Hao, Fenglin Deng, Guang Chen, Zhong-Hua Chen, Fanrong Zeng, Yuan Qin, Wei Jiang

**Affiliations:** 1 MARA Key Laboratory of Sustainable Crop Production in the Middle Reaches of the Yangtze River (Co-Construction by Ministry and Province), Hubei Key Laboratory of Waterlogging Disaster and Agricultural Use of Wetland, College of Agriculture, Yangtze University, Jingzhou 434025, China; 2 China National Rice Research Institute, Hangzhou 311401, China; 3 Guangdong Academy of Sciences, Guangzhou 510316, China; 4 School of Agriculture, Food and Wine, Waite Research Institute, The University of Adelaide, Adelaide, SA 5064, Australia; 5 Xianghu Laboratory, Hangzhou 311231, China; 6 College of Agriculture, Nanjing Agricultural University, Nanjing 210095, China

**Keywords:** copper homeostasis, COPT, detoxification, plants, transcriptional regulation

## Abstract

Copper (Cu) is an essential micronutrient for plants, playing a crucial role in various physiological and molecular processes. Excess Cu induces oxidative stress and disrupts cellular functions, while Cu deficiency causes chlorosis and poor pollen development, thereby reducing crop yields. However, the molecular and evolutionary mechanisms of Cu tolerance and homeostasis remain unclear in the plant kingdom. In this review, we discuss the uptake, transport, and detoxification of Cu through high-affinity Cu transporters (COPTs). Additionally, we update recent studies on maintaining Cu balance by mediating the root exudation of organic acids (e.g., citrate and proline), xylem/phloem loading, cell wall binding, vacuolar sequestration, redistribution, and the activity of antioxidant enzymes (e.g., SOD, CAT, and APX). Furthermore, tissue-specific expression analyses reveal that *COPT* genes exhibit distinct spatial regulation in the roots and leaves, which are the primary sites of Cu transport and detoxification. Overall, our review highlights the critical roles of *COPT* gene families and detoxification pathways in maintaining Cu homeostasis in plants. Future research should focus on genetic engineering approaches to enhance Cu tolerance, optimize Cu distribution in grains, and mitigate soil contamination risks. By clarifying these mechanisms, we can develop strategies to sustain crop production under increasing Cu stress, thereby ensuring food security and human health.

## 1. Introduction

Copper (Cu) is a vital trace element for living organisms, and it must be maintained within a dynamic equilibrium in the plants [1]. Furthermore, Cu functions as a cofactor for numerous proteins and enzymes, which are involved in distinct physiological and biochemical processes, including photosynthesis, respiration, protein transport, cell wall metabolism, antioxidant defense, plant hormone signal transduction, and disease resistance [2,3,4,5,6,7]. However, either an excess or a insufficiency of Cu in the soil has been demonstrated to disrupt Cu homeostasis in plants growing in soil. In recent years, excessive mining, industrial particulate emissions, and the widespread application of Cu-containing pesticides [8] have contributed to the accumulation of Cu in the air, soil, and water, which cause serious environmental pollution and crop (e.g., rice, maize, and wheat) yield reduction [1].

Both Cu deficiency and Cu excess have the capacity to affect plant growth and development [1,9]. When Cu levels in plant aerial tissues are below 5 mg/kg or above 20 mg/kg, significant symptoms of Cu deficiency or Cu toxicity will be observed [5,6]. A deficiency of Cu in plants can result in a number of adverse effects, including reduced growth rate, chlorosis of young leaves, leaf margin curling, apical meristem damage, and impaired cell wall biosynthesis. Collectively, these disruptions compromise water transport efficiency, hinder pollen development, and ultimately diminish crop yield [6,10]. In contrast, excessive Cu might trigger a series of redox reactions, thereby generating excessive reactive oxygen species (ROS) and free radicals within plant cells [8,11]. These oxidative byproducts subsequently degrade membrane integrity, suppress photosynthetic capacity, perturb enzymatic functions, and inhibit plant growth, in addition to inducing broader physiological damage [12]. Notably, Cu deficiency in humans is associated with osteoporosis, anemia, and developmental impairments, whereas dysregulated Cu metabolism underlies the pathogenesis of Menkes disease and Wilson disease [13,14]. In order to combat these challenges, plants have evolved a sophisticated transport system to sense Cu and precisely modulate Cu uptake and accumulation [15]. Given these complexities, it is critical to understand the mechanisms of uptake, transport and distribution of nutrients in plants, as well as the regulation of their accumulation and dynamic balance. A comprehensive understanding of these processes is essential for ensuring normal plant growth and development, as well as human nutritional health [16,17].

Numerous vital proteins involved in Cu tolerance have been identified and extensively characterized across diverse plant species. For instance, Cu transporters (COPT/CTR) function as the critical gateway for Cu uptake and as the central hub for inter-organ Cu distribution in plants, playing an indispensable role in maintaining cellular Cu homeostasis. To date, *COPT* family members have been identified in a wide range of plant and fungi species, including *Zea mays* [18], *Solanum lycopersicum* [19], *Pleurotus ostreatus* [20], *Kandelia obovate* [21], *Medicago sativa* [22], *Lotus japonicus* [23], and *Physcomitrium patens* [24]. Moreover, their functions in response to Cu uptake and transport have also been explored, such as in *Oryza sativa OsCOPT7* [25,26,27] and *Medicago truncatula MtCOPT1* [28]. To adapt to the adverse Cu supply environment, plants have evolved a more adaptive Cu utilization system in terms of Cu absorption, transport, and distribution. Beyond the well-characterized COPT family [19,20], multiple transporter families contribute to Cu homeostasis, including heavy-metal P1B-type ATPases (HMAs) [29], ZRT/IRT-related transporters (ZIPs) [30], yellow stripe-like proteins (YSLs) [31], and natural resistance-associated macrophage proteins (NRAMPs) [32]. While the molecular and physiological mechanisms underlying plant responses to Cu stress have been well documented [1,8], the evolutionary divergence and functional diversification of *COPTs* across the plant kingdom remain poorly understood.

In this review, we focused on the phylogenetic relationships of the *COPT* gene families from lower to higher plants, with a comprehensively investigation of their expansion in green plants. Furthermore, we conduct comparative analyses of their tissue-specific expression profiles across bryophytes, gymnosperms, and angiosperms. Additionally, we also analyzed the differential responses of *COPT* genes to varying Cu supply levels in representative monocot (*O. sativa*) and eudicot (*Arabidopsis thaliana*) species. Collectively, this review not only elucidates the evolutionary trajectory and functional specialization of *COPT* genes but also provides novel insights into the regulatory mechanisms plants employ to maintain Cu homeostasis under both deficient and excessive conditions.

## 2. Soil Copper Contamination: Sources, Ecological Risks, and Crop Toxicity

In the soil, Cu exists in various chemical forms, including oxides (e.g., Cu_2_O and CuO), sulfates, sulfides, carbonates, and native Cu [33,34]. Notably, Cu is typically present within the lattice of primary and secondary minerals, which exhibits a higher affinity for soil organic matter in comparison to that of divalent metals such as nickel (Ni), lead (Pb), cobalt (Co), zinc (Zn), manganese (Mn), and magnesium (Mg) [35]. Under natural conditions, the average concentration of Cu in the soil varies between 6 and 80 mg/kg [36]. However, in recent years, the extensive utilization of Cu-containing fungicides in agricultural production, in conjunction with heightened industrial emissions of the “three wastes” (waste gas, water, and solids), has resulted in elevated levels of high Cu content in soil [37]. Critically, unlike organic pollutants, accumulated Cu resists microbial and chemical degradation, thereby posing persistent threats to ecosystem integrity, food security, and public health [38,39]. According to China’s 2014 National Soil Pollution Survey Bulletin, 16.1% of surveyed soils exhibited contamination, with Cu exceeding standards in 2.1% of cases. Furthermore, analysis of soil samples from 102 Cu mines revealed that the concentrations of Cu and cadmium (Cd) in the soil exceed moderate to severe pollution levels in regions globally [40]. Compared with other regions, the pollution levels were higher in Oman, China, Australia, and the United Kingdom [40]. These soil heavy metals also induced a high ecological risk, with Cu contributing 21.7% [41]. It is evident that Cu has become an significant heavy-metal soil pollutant in numerous regions, which is becoming increasingly prominent [42].

**Figure 1:**
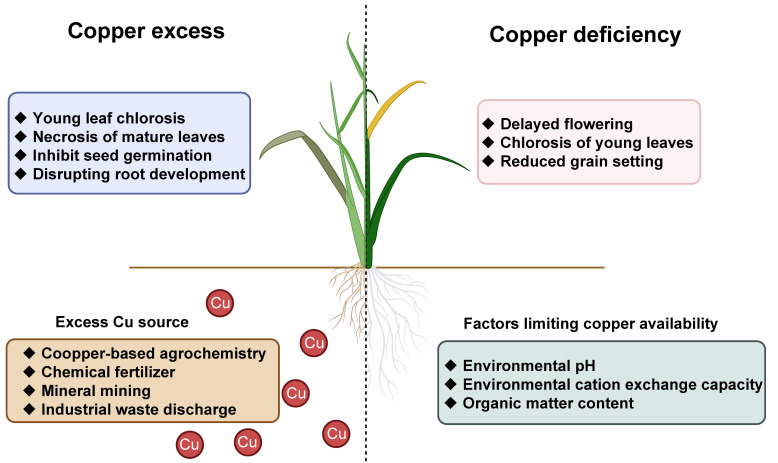
Schematic diagram of environmental factors influencing Cu levels and the disruption of Cu homeostasis. On the left are the sources of excess-Cu environments and the symptoms of Cu toxicity in plants, and on the right are the factors affecting Cu bioavailability and the symptoms of Cu deficiency in plants. This figure was drawn using BioRender website (https://app.biorender.com/ [accessed on 28 June 2025)]).

Once soil is polluted with Cu, the excess Cu may be absorbed by plant roots and cause toxicity to the plants [43]. As a result, excessive Cu in the soil has become one of the major limiting factors for crop yield and quality [44]. This is particularly evident in rice (*O. sativa*), one of the most important food crops in the world, which demonstrates marked susceptibility to Cu toxicity [34]. For instance, controlled greenhouse experiments conducted in 2019 and 2021 revealed that the yield of Wufeng You 286 decreased by 31.2% and 39.5%, respectively, in soil contaminated with 200 mg/kg of Cu compared to unpolluted soil [45]. The median toxicity concentration order of trace metals in hydroponic nutrient solutions was reported to be Pb (0.30 μM) ≈ Hg (0.47 μM) > Cu (2.0 μM) > Cd (5.0 μM) ≈ arsenate [As (V)] (9.0 μM) > Co (17 μM) ≈ Ni (19 μM) ≈ Zn (25 μM), indicating that Cu’s phytotoxicity surpasses that of most heavy metals except Pb and Hg [46]. Given these impacts, by elucidating the molecular mechanisms of Cu excess and deficiency in crops (Figure 1), our study is of great significance in improving Cu tolerance and enhancing yield and quality in crops [1].

## 3. CTR/COPT Copper Transporters: Structural Conservation and Functional Diversification

The first step of Cu homeostasis is the transmembrane transport of Cu from the soil or solution into the plant cell. In the process, the CTR/COPT family members serve as the main proteins regulating Cu absorption. These transporters are widely present in eukaryotes and are known as CTR in fungi and animals and COPT in plants [47,48,49]. CTR/COPT proteins are generally localized on the cell membrane and have three conserved transmembrane domains (TMDs) that are rich in the amino acid methionine (Met). The amino terminus of these proteins is localized outside of the cell, while the carboxyl terminus is localized inside of the cytoplasm. Importantly, the Met motif in the extracellular amino terminus plays a key role in recognizing and binding Cu ions [50]. However, this family exhibits functional diversification across different species, ranging from basic Cu uptake to tissue-specific transport in plants. This “structural conservation-functional diversification” pattern not only reflects the evolutionary constraints on Cu transport mechanisms but also highlights species-specific adaptations.

### 3.1. Functional Diversification of CTR/COPT Copper Transporters Across Green Plants and Yeast

In yeast (*Saccharomyces cerevisiae*), three members of the CTR transporter family have been identified: ScCtr1 to ScCtr3. Among these, ScCtr1 was the first CTR protein to have been identified as being involved in Cu absorption [21]. Interestingly, under excess-Cu conditions, the *scctr2* yeast mutant exhibited higher tolerance, whereas overexpression of *ScCtr2* increases sensitivity to excess Cu treatment [51]. Moreover, both ScCtr1 and ScCtr3 could function independently in Cu absorption, and the inactivation of *ScCtr1* and *ScCtr3* significantly reduces Cu absorption in yeast [52].

In *A. thaliana*, there are six members of the COPT family, which are named AtCOPT1–AtCOPT6. AtCOPT1 is localized on the cytoplasmic membrane and plays a significant role in Cu absorption in root and pollen development [53]. Likewise, AtCOPT2 is also localized on the cytoplasmic membrane, and has the highest expression levels in the roots of *A. thaliana*, playing a crucial role in Cu absorption and distribution [53]. Additionally, Cu deficiency significantly increases the expression levels of *AtCOPT1* and *AtCOPT2*. In contrast, AtCOPT3 is also localized on the cytoplasmic membrane and is mainly expressed in pollen grains and vascular bundles, which are involved in the transport of Cu from inside the cell to outside. Consistent with this function, the *atcopt3* mutant exhibited changes in pollen morphology [23]. However, AtCOPT4 cannot transport Cu in the yeast mutants and its specific function remains unclear. On the other hand, AtCOPT5 is localized on the vacuolar membrane, which releases Cu ions stored in vacuoles into the cytoplasm under Cu deficiency [54]. This process supplies Cu when plants require it during the processes of growth and development. The *atcopt5* mutant showed inhibited root and shoot growth, as well as reduced chlorophyll content under Cu deficiency [54]. Furthermore, AtCOPT6 was localized on the cell membrane, and its expression level was increased by the deficiency in Cu. Under such conditions, the *atcopt6* mutant displayed increased Cu accumulation in rosette leaves and reduced Cu content in seeds. These findings indicate that AtCOPT6 may play a role in Cu distribution in the aboveground part and Cu transport in seeds [55].

In *O. sativa*, seven COPT proteins have been identified and designated as OsCOPT1–OsCOPT7 [27]. Structurally, all of them contain three TMDs with no introns, and they exhibit 35–64% sequence homology and 47–73% sequence similarity amongst themselves [52]. Functionally, OsCOPT1 and OsCOPT5 are localized on the cytoplasmic membrane, and their expression levels increase under Cu deficiency, while excess Cu levels inhibit their transcription. Overexpression of *OsCOPT1* or *OsCOPT5* increases Cu content in the roots and shoots of plants but reduces Cu content in xylem sap. Conversely, knockout of *OsCOPT1* or *OsCOPT5* in rice showed an opposite effect, indicating that OsCOPT1 and OsCOPT5 are involved in Cu transport and distribution in rice [7]. Interestingly, the expression patterns of *OsCOPT2* and the transcription factor *OsMYB84* are similar, with exogenous Cu inducing their expression levels and Cu deficiency inhibiting their transcription. *OsMYB84* can promote Cu absorption by upregulating the expression of *OsCOPT2* [56]. In heterologous expression studies, OsCOPT2, OsCOPT3, and OsCOPT4 showed no Cu transport activity alone, but when co-expressed with OsCOPT6, they were shown to be able to mediate Cu absorption in the Cu absorption-deficient yeast *ctr1Δctr3Δ* mutant [52]. Furthermore, the heterologous expression of *OsCOPT6* in the yeast *S. cerevisiae* mutant has been demonstrated to restore the Cu absorption-deficient phenotype of the *ctr1Δctr3Δ* mutant, demonstrating that OsCOPT6 is involved in Cu transport. In conditions of normal Cu supply, the expression of *OsCOPT6* is predominantly observed in the upper region, whereas Cu deficiency significantly increases its expression level, and excess Cu inhibits it [52]. Currently, the functions of COPT family members in Cu accumulation and antiviral responses have been investigated. For instance, under normal growth conditions, the *oscopt7* mutant was observed to demonstrate a higher Cu concentration in the shoot, in comparison with the wild type [57]. Additionally, OsCOPT7 has been reported to be localized on vacuolar membranes and the endoplasmic reticulum [26], and its expression is upregulated in response to Cu deficiency [57]. Interestingly, knockout of *OsCOPT7* increased Cu accumulation in roots [57] but reduced the Cu concentration in the shoots of the plant and grains. These results indicate that OsCOPT7 is responsible for exporting Cu from the vacuoles and endoplasmic reticulum, playing a crucial role in transporting Cu from the roots to the shoots in the *O. sativa*. Although the Cu transport activity of all members of the OsCOPT protein family has been confirmed in heterologous systems [52], the specific functions of these proteins in Cu absorption, transport, and distribution in *O. sativa*, as well as their regulatory mechanisms, remain unclear.

Despite functional differentiation, the Cu transport capacity remains highly conserved across plant species. For example, in *Brachypodium distachyon*, BdCOPT3 and BdCOPT4 are localized on the cytoplasmic membrane, and their expression levels in roots and leaves increase significantly under Cu deficiency [58]. Furthermore, *MtCOPT1* is the only root nodule-specific *COPT* gene that can transport Cu from the apoplast to root nodule cells. This provides Cu for the metabolic processes necessary for symbiotic nitrogen fixation [28]. In yeast heterologous expression systems, *Lotus* COPT proteins can restore growth in Cu-deficient yeast, indicating their role in Cu absorption [23]. Likewise, when *ZmCOPT1-3* from *Z. mays* was introduced into the Cu-deficient yeast strain, the expression of ZmCOPT in the yeast strains significantly alleviated the growth inhibition caused by Cu deficiency [18]. In *S. lycopersicum*, SlCOPT1 and SlCOPT2 have been shown to effectively restore the growth ability of the defective yeast, while SlCOPT3 and SlCOPT5 have only been demonstrated to restore it to a slight extent. However, SlCOPT6 has been demonstrated to be incapable of restoring the growth defect of the Cu-deficient yeast [19]. Similarly, PpaCOPT1 and PpaCOPT2 in *P. patens*, which are localized to the tonoplast and plasma membrane, respectively, have been demonstrated to be capable of mitigating the growth defects of the defective yeast under low Cu levels [24]. Overall, these studies highlight the conserved yet diverse roles of COPTs in maintaining Cu homeostasis across different plant species. However, further research is needed to elucidate the precise regulatory mechanisms and functional specificities of these proteins.

### 3.2. Evolutionary Conservation of COPT Copper Transporters Across Green Plants

**Figure 2:**
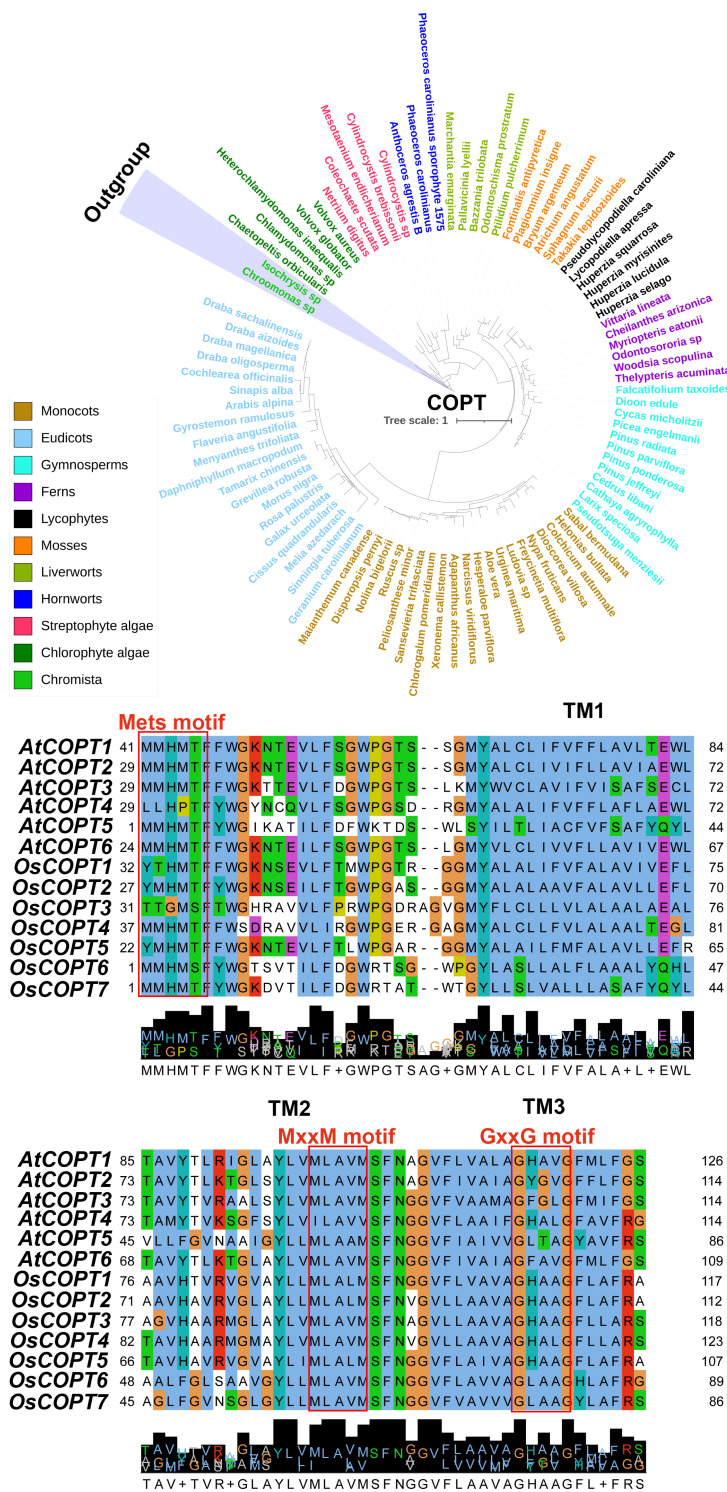
Evolutionary analysis of COPT proteins in land plants and algal species. (**A**) A phylogenetic tree including species in major clades in eudicots, monocots, gymnosperms, ferns, lycophytes, mosses, liverworts, hornworts, and algae. Motif alignment (**B**) of COPT proteins in *Arabidopsis thaliana* and *Oryza sativa*.

**Figure 3:**
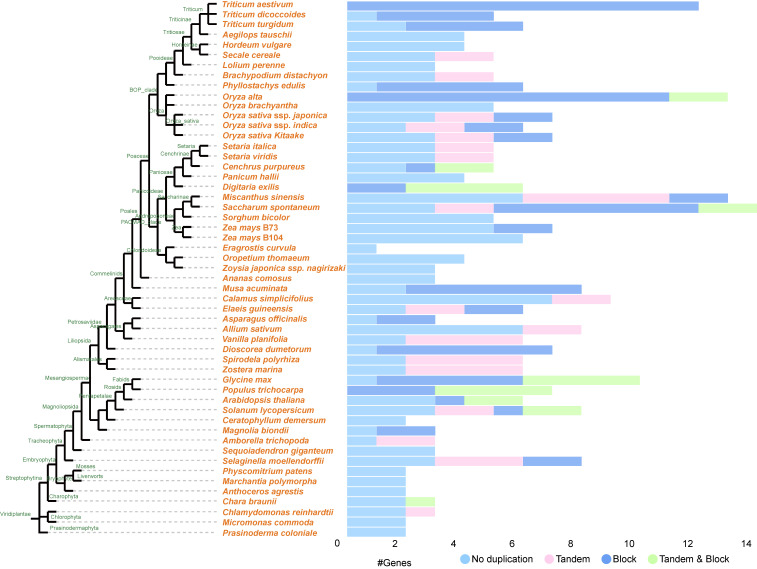
Tandem and block gene duplicate of *COPT* gene family in *Chlorophyta* and *Embryophyta* (https://bioinformatics.psb.ugent.be/plaza.dev/instances/monocots_05/genes/gene_duplication_analysis/interpro/IPR007274 [accessed on 21 May 2025)]). All gene numbers were download from the PLAZA database (https://bioinformatics.psb.ugent.be/plaza/ [accessed on 21 May 2025)]) containing >100 plant and algal species. The phylogenetic tree of distinct species was obtained through TimeTree (http://www.timetree.org/ [accessed on 21 May 2025)]).

The homologues of COPT from representative plant and algal species were identified through BLASTP tool searches from the OneKP database (https://db.cngb.org/blast/blast/blastp/ [accessed on 21 June 2025)]) according to our previous studies [59,60,61,62]. Phylogenetic analysis revealed the widespread distribution of COPTs in green plants. Interestingly, COPTs can be traced back to *Chlorophyta* (green algae) species at the earliest, including *Chaetopeltis orbicularis* and *Volvox globator* (Figure 2A). The *COPT* family has been identified in many plants such as *A. thaliana*, *O. sativa*, *Populus trichocarpa*, an *Vitis vinifera*, and most of their members possess three transmembrane domains: TMD1, TMD2, and TMD3 [21]. Conserved motifs, including Mets-motifs, the MxxxM motif, and the GxxxG motif of COPT members in most green plants [18,52,63], were identified in COPTs of *A. thaliana* and *O. sativa* (Figure 2B). The Ctr copper transporter gene family has 294 genes in the 53 examined species of Chlorophyta and Embryophyta, and the percentages of tandem and blockin this gene family were 16% and 30%, respectively (Figure 3). When the two methionines in the MxxxM motif are mutated, COPT loses its Cu transport function, indicating that the MxxxM motif is a critical structure for Cu transport [64]. Genetic, biochemical, and structural data suggest that extracellular methionine-rich motifs help Cu^+^ enter the cell by attracting it to the pore entrance [65]. The function of Mets-motifs across different species may need to be further explored through biochemical and structural evidence. Consequently, COPTs exhibit significant conservation among green plants with regard to molecular evolutionary aspects, playing an indispensable role in Cu uptake and transport. However, when BLASTP is used to align highly divergent sequences, the results may have low sequence similarity. This can lead to inaccurate alignments or the omission of important homologous relationships. Future work could involve exploring the use of more advanced alignment tools to refine these analyses further.

## 4. Expression Analysis of COPT Genes in Diverse Plants

### 4.1. Tissue-Specific Gene Expression Analysis of COPT Genes

**Figure 4:**
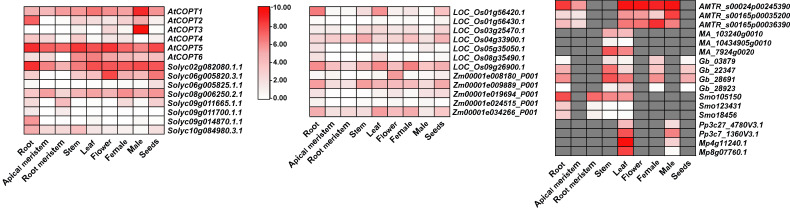
Expression of *COPT* genes of diverse tissues and organs (male portion, female portion, apical meristem, root meristem, flower, seed, root, leaf, and stem) in eudicots (*A. thaliana*, *S. lycopersicum*), monocots (*O. sativa*, *Z. mays*), basal angiosperms (*A. trichopoda*), gymnosperms (*P. abies*, *G. biloba*), lycophytes (*S. moellendorffii*), moss (*P. patens*), and liverworts (*M. polymorpha*). Data were downloaded from a public database (https://evorepro.sbs.ntu.edu.sg/heatmap/comparative/tree/41304/raw [accessed on 25 May 2025)]).

The expression of *COPT* genes in response to Cu stress has been documented in model plants [1]. Nonetheless, the expression in the other plants remains unclear. Here, in *A. thaliana*, *O. sativa*, *Z. mays*, *S. lycopersicum*, *Amborella trichopoda*, *Picea abies*, *Gingko biloba*, *Selaginella moellendorffii*, *Physcomitrium patens*, and *Marchantia polymorpha*, the transcript levels of *COPTs* among the representative species were analyzed in various tissues and organs, including the root, flower, leaf, stem, female portion, seeds, male portion, apical meristem, and root meristem (Figure 4).

Most *COPT* genes showed high expression in the roots and leaves. Compared to other species, the *COPT* genes from *A. thaliana AtCOPT* (except *AtCOPT3*) and *O. sativa OsCOPT* (except *LOC_Os01g56430.1*, *LOC_Os05g35050.1* and *LOC_Os08g35490.1*) showed high expression across a wide range of tissues and organs. Specailly, *A. thaliana AtCOPT5*, *S. lycopersicum Solyc02g082080.1.1*, *A. trichopoda AMTR_s00024p00245390*, *S. moellendorffii Smo105150*, and *LOC_Os01g56420.1* were highly expressed in the roots. Furthermore, *AtCOPT1*, *AtCOPT5*, *Solyc02g082080.1.1*, and *LOC_Os09g26900.1* showed consistently high expression in all examined tissues and organs. Notably, *AMTR_s00024p00245390* had extremely high expression in leaves and flowers. However, *P. abies MA_103240g0010*, *MA_10434905g0010*, and *MA_7924g0020* were only expressed in the stems and leaves, indicating their functional similarity. Interestingly, *P. patens Pp3c27_4780V3.1*, *Pp3c7_1360V3.1*, and *M. polymorpha Mp4g11240.1* exhibited specific expression levels in leaves and male portions. It is noteworthy that the male portion in *P. patens* and *M. polymorpha* refers specifically to the male gametophyte. The expression patterns of different *COPT* genes might imply the conservation and divergence of their functions across diverse plant species.

### 4.2. Single-Cell Expression Analysis of COPT Genes

**Figure 5:**
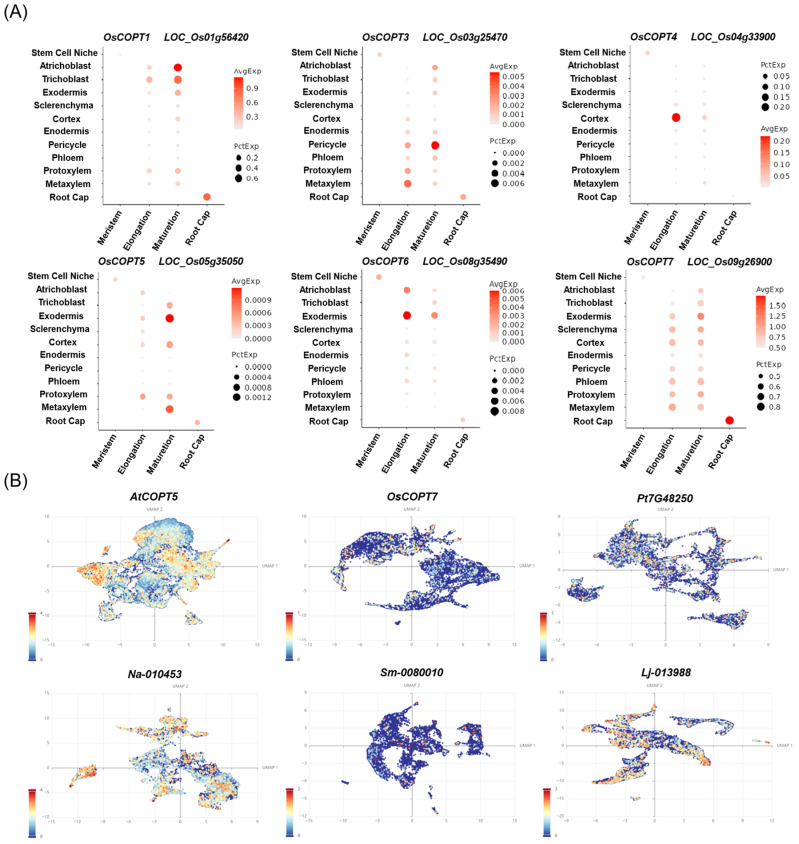
Single-cell expression analysis of *COPTs*. Data were downloaded from public databases (https://rice-singlecell.shinyapps.io/orvex_app/ [accessed on 10 July 2025)] and http://shoot.plantcellatlas.com) [accessed on 30 July 2025)]. (**A**) Single-cell expression analysis of *COPTs* in the root tips of *O. sativa*. (**B**) Single-cell expression analysis of *AtCOPT5* (*A. thaliana*), *OsCOPT7* (*O. sativa*), *Pt7G48250* (*P. tabuliformis*), *Na-010453* (*N. auriculata*), Sm-0080010 (*S. martensii*), and Lj-013988 (*L. japonicum*).

Root systems are essential organ for plants to acquire mineral nutrients, and the gene expression of each cell type directly determines the plant’s capacity for mineral element uptake. With the development of single-cell technology, the gene expression profile at the single-cell level of the *O. sativa* root system has been studied [66,67]. In previous studies, the *O. sativa* root system was divided into 12 cell types at the cellular level, including the stem cell niche, artichoblast, trichoblast, exodermis, sclerenchyma, cortex, enodermis, pericycle, phloem, protoxylem, metaxylem, and root cap [66]. Using a public database (https://rice-singlecell.shinyapps.io/orvex_app/ [accessed on 10 July 2025)]), we analyzed the single-cell level of the *OsCOPT* family. It was found that only *OsCOPT2* did not detect expression in the root system among the seven members, while *OsCOPT1* and *OsCOPT7* were detected in most cell types of the *O. sativa* root system (Figure 5 and Figure S1). Moreover, their expression had a strong preference in diverse cell types, with *OsCOPT1* having the highest expression in the mature zone of atrichoblasts, *OsCOPT3* in the pericycle of the mature zone, *OsCOPT4* in the cortex of the elongation zone, *OsCOPT5* in the exodermis of the mature zone, *OsCOPT6* in the exodermis of the elongation zone, and *OsCOPT7* in the root cap cells (Figure 5). Furthermore, these genes recently showed similar expression in another database (http://www.elabcaas.cn/scmr/index.html, accessed on 24 July 2025), but this database contains data on more tissues. Thus, different genes have distinct expression patterns in different cells. Remarkably, single-cell RNA sequencing analysis applied to the shoot apices of six distinct vascular plant species (including *A. thaliana*, *O. sativa*, *Pinus tabuliformis*, *Nephrolepis auriculata*, *Selaginella martensii*, and *Lycopodium japonicum*) facilitated the assembly of a high-fidelity single-cell atlas. In this single-cell study encompassing six species of vascular plants, the annotated cell populations comprised epidermal cells (ECs), mesophyll/cortex cells (MCs), vascular tissue cells (VCs), and proliferating cells (PCs) in the S or G2/M phases [68]. We used OsCOPT7, characterized to play a critical role in Cu homeostasis in rice, as the query to identify its closest homologs via BLAST analysis in five other vascular plant species. The resulting orthologs, namely AtCOPT5, Pt7G48250, Na-010453, Sm-0080010, and Lj-013988, exhibited sequence similarities ranging from 45% to 57% (Figure 5B). Moreover, they all exhibited the highest expression levels in vascular cells, indicating high functional similarity among them. This analysis revealed the evolutionarily conservation of COPT proteins at the single-cell level. This also implies that single-cell analyses may need to be extended to a broader range of species, thereby providing more comprehensive guidance for functional studies of genes.

### 4.3. Gene Expression Analysis of COPT Genes in a Eudicot and a Monocot Under Conditions of Cu Excess or Deficiency

**Figure 6:**
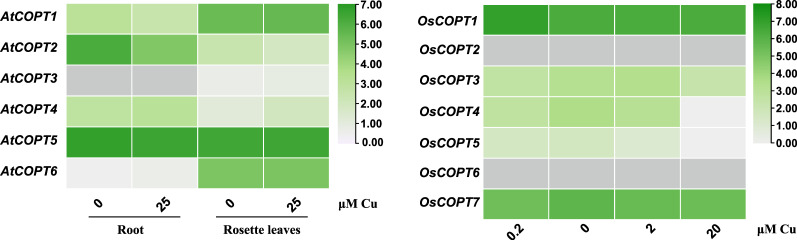
Expression analysis of *COPT* genes’ response to different Cu concentrations in eudicots and monocots. (**A**) Expression of *AtCOPT* genes in roots and rosette leaves. (**B**) Expression of *OsCOPT* genes in roots.

In addition to the tissue-specific analysis of genes encoding Cu transporter proteins, we also performed response expression analysis of *COPT* genes to study the response to Cu-induced stress in monocot (*O. sativa*) and eudicot (*A. thaliana*) model plants (Figure 6). In the roots of *O. sativa*, the expression of *OsCOPT3*, *OsCOPT4*, and *OsCOPT7* are induced to a slight extent by Cu deficiency, while *OsCOPT1*, which has the highest expression level, is slightly repressed by Cu deficiency. Interestingly, our results are generally in agreement with those of previous studies [27]. In the roots of *A. thaliana*, expression of *AtCOPT1*, *AtCOPT2*, and *AtCOPT5* was strongly upregulated in response to Cu deficiency, whereas in rosette leaves, only *AtCOPT2* exhibited significant induction under the same conditions. Moreover, *AtCOPT4* expression was uniformly repressed by Cu deficiency in both organs. Notably, *AtCOPT6* transcript levels remained largely unresponsive to varying Cu availability in either roots or rosette leaves. These tissue expressions indicated that there are differences in the response to Cu stress between monocots and eudicots.

## 5. Cu Transport Systems and Cu Chaperone Proteins in Plants

**Figure 7:**
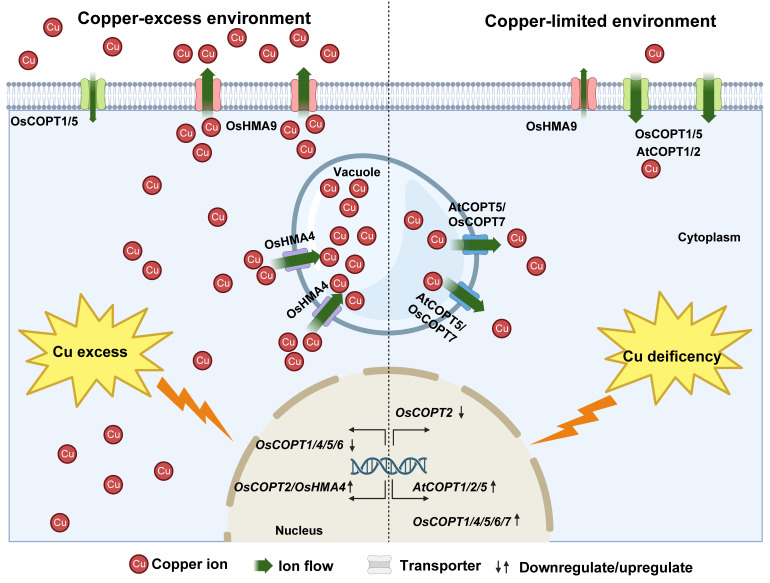
Proposed model of the transport system of Cu tolerance. On the left is a excess-Cu environment, and on the right is a Cu-limited environment. COPTs, copper transporters; HMA, heavy-metal P1B-type ATPases. This figure was drawn using BioRender (https://app.biorender.com/ [accessed on 28 June 2025)]).

Cu is essential for the growth and development of plants, and its absorption is an energy-consuming active transport process [35]. Despite the abundance of Cu in soil, its strong binding affinity for soil organic matter limits the concentration of Cu available for plant uptake [69]. Once soil is contaminated with Cu, excessive Cu will accumulate in plants. Both Cu deficiency and excess are harmful to plant growth and development. The absorption, transport, and redistribution of Cu in plants involve multiple transporters to maintain Cu homeostasis (Figure 7).

In order to respond to changes in external Cu levels and maintain a balanced supply of Cu in the plant, higher plants have evolved a sophisticated and stringent Cu homeostasis regulation system [70,71]. Cu^2+^ in the environment is initially reduced to Cu^+^ by Cu^2+^-reductases (AtFRO4/AtFRO5) localized on the plasma membrane [69]. Subsequently, the absorbed Cu^+^ is transported into the plant body through high-affinity Cu transporters in the roots. At the cellular level, these processes are mediated by interactions between Cu chaperone protein families and Cu transporter protein families, which maintain the balance required for plant growth and development for preventing Cu poisoning [72]. At the tissue level, the transport of Cu necessitates root absorption, vacuolar sequestration, and loading through the xylem and phloem. The process further entails allocation and redistribution at the plant nodes via diverse transporters [70,73]. For instance, the Cu chaperone proteins (CCHs) can be divided into three categories: antioxidant proteins (antioxidant-like proteins, ATX-like), Cu chaperones for SOD (CCS), and cytochrome oxidases (COXs). Furthermore, Cu transporters can be classed into two categories: the first category transfers Cu from outside the cell to the inside (influx); the second category is responsible for expelling Cu from the inside of the cell to the outside or transporting it to specific organelles (efflux). In addition to high-affinity Cu transporters (COPTs), members of the HMA, ZIP, NRAMP, and YSL families have been shown to play a role in Cu transport [8].

HMA proteins are found in a variety of plant species and play a role in regulating metal ion homeostasis, including that of Cu ions [74]. Among these transporters, the chloroplast envelope-localized AtHMA1 functions to transport excess Zn^2+^ and Cu^2+^ from chloroplasts into the cytoplasm, thereby reducing their potential damage to the photosynthetic system. Notably, in the *AtHMA1* knockout mutant, the Cu content of the plant was significantly reduced, highlighting its importance in Cu homeostasis [75]. Similarly, AtHMA6/PAA1 is also localized on the chloroplast envelope, where it specifically facilitates the transportation of Cu^+^ into the chloroplasts. This process is essential for the provision of the necessary cofactors for plastocyanin in the thylakoid lumen and Cu-Zn superoxide dismutase (Cu/Zn SOD) [76]. In contrast, the loss of function of plasma membrane-localized AtHMA5 resulted in increased Cu sensitivity and higher Cu accumulation in the roots compared to the WT [76]. Furthermore, the athma7/ran1 mutant demonstrated higher sensitivity to Cu deficiency, as evidenced by its suppression of cell growth and expansion. Importantly, exogenous Cu supplementation was found to partially restore the mutant phenotypes [77]. Additionally, AtHMA8/PAA2, localized in the thylakoid membrane, plays a specialized role in transporting Cu from the chloroplast stroma to the thylakoid lumen, where it is incorporated into plastocyanin [78,79].

ZIPs are a class of metal transporters that have been identified in a variety of plant species, with particular prevalence in dicots such as Arabidopsis. The primary function of these proteins is the transportation of various metal cations into the cytoplasm, including but not limited to Zn^2+^, Mn^2+^, Fe^2+^/Fe^3+^, Cd^2+^, Co^2+^, Ni^2+^, and Cu^2+^. Research has demonstrated that AtZIP2 and AtZIP4 have the capacity to ameliorate the growth defects exhibited by Cu and Zn transport mutants in yeast [80].

NRAMPs are a class of metal transporters found in plants, facilitating the absorption, distribution, and sequestration of metallic elements within various plant tissues [32]. The expression of OsNRAMP2 is significantly induced by Cu deficiency; however, the role of OsNRAMP2 in Cu transport remains to be elucidated [81]. Furthermore, OsNRAMP5 was possibly involved in the uptake and transportation of Cu [82]. Interestingly, all five NRAMP members in *Kandelia obovata* showed upregulation or downregulation in response to Cu deficiency or excess [32]. Nevertheless, the precise roles of these proteins in Cu transportation are yet to be elucidated and necessitate additional research to ascertain their specific functions.

YSL transporters have been identified in a variety of higher plants and have been implicated in the long-distance transport of metal ion chelates, such as nicotianamine (NA), and in the long-distance transport of phytosiderophores (Ps) [83]. The Arabidopsis YSL family comprises eight members, three of which (AtYSL1, AtYSL2, and AtYSL3) are localized in the cytoplasmic membrane and are involved in transporting Cu-NA, thereby affecting Cu distribution in plants [84,85]. Furthermore, OsYSL16 is capable of transporting Cu–nicotianamine (Cu–NA) complexes. Plants lacking OsYSL16 function show increased Cu concentrations in older leaves but decreased Cu concentrations in younger leaves, indicating a defect in the remobilization of Cu from older to younger leaves [86].

Cu chaperone proteins, in conjunction with the HMA, COPT, ZIP, and YSL transporters, constitute a system that maintains Cu homeostasis in plants, thereby providing a robust foundation for plant adaptation to complex environments. Nevertheless, the evolutionary mechanisms of Cu transporters and their cognate chaperones, as well as the molecular logic governing their combinatorial control of systemic Cu homeostasis in plants, remain outstanding questions that warrant comprehensive investigation.

## 6. Plant Cu Detoxification and Tolerance Mechanisms

Excessive absorption of Cu into plant cells induces the accumulation of ROS, which subsequently impairs plant growth and development. To adapt to environments with excess Cu, plants have evolved multiple detoxification and tolerance mechanisms. Primarily, these protective strategies include the following: (i) root exudate-mediated chelation, (ii) cell wall binding and vacuolar sequestration, (iii) active metal efflux, and (iv) induction of antioxidant enzymes [1]. Importantly, these coordinated processes maintain an optimal balance between cellular Cu levels and ROS production, thereby effectively alleviating Cu toxicity [87,88]. The response patterns of the COPT and HMA transport systems to Cu excess and deficiency have been characterized in *O. sativa* and *A. thaliana* (Figure 7).

### 6.1. Root Exudates

Plant root exudates (e.g., organic acids and amino acids) have the capacity to influence the availability and mobility of heavy metals in the soil. This, in turn, can affect the uptake and translocation of heavy metals by plants. The effects of the alterations in plant root exudates on the soil include changes in pH and redox potential (Eh). Furthermore, these exudates chelate heavy metals and enhance microbial activity [89]. In the presence of excess Cu, plant roots have been observed to secrete organic acids, including citric acid, oxalic acid, malic acid, tartaric acid, and succinic acid. These acids have the capacity to bind with heavy metals, forming nontoxic complexes. Additionally, they have been shown to mitigate the toxicity of heavy metals to plants by promoting plant growth and enhancing the activity of antioxidant enzymes [90]. In *Phyllostachys pubescens*, Cu has been observed to induce the secretion of low-molecular-weight organic acids, including oxalic acid, malic acid, and lactic acid, from the plant’s roots [91]. Amino acids, such as proline, contain functional groups, including amino, carboxyl, and hydroxyl groups. These functional groups can bind with heavy metals to form stable compounds, thereby achieving the objectives of detoxification and immobilization. When applied externally, proline can reduce the production of ROS in wheat (*Triticum aestivum*) under Cu stress and improve photosynthetic efficiency [92]. In *Brassica napus*, the concentration of free amino acids, such as proline, cysteine, alanine and aspartic acid, increases as the Cu concentration rises in the seedlings [93]. Additionally, the exogenous addition of β-amino butyric acid (BABA) enhances Cu tolerance by regulating the content of ROS and the activity of related antioxidant enzymes in tobacco [94]. Under Cu stress, the induced secretion of organic acids is likely to be strongly associated with heavy metal detoxification; however, the specific molecular mechanisms through which this occurs still need to be further explored.

### 6.2. Isolation and Compartmentalization

The strategies of plants response to Cu stress include isolating absorbed metal ions in metabolically inactive tissues, organs or subcellular structures such as epidermal cells, vascular bundles, cell walls, and vacuoles [95]. Among these, cell walls and vacuoles represent the most important locations for Cu binding and sequestration [96]. The primary locations for the binding of Cu in roots are cell walls and the vacuoles. The cell wall fulfills the function of a barrier that prevents metal ions from penetrating the cell membrane and entering the cytoplasm [97]. The cell wall is composed of pectin, cellulose, hemicellulose, and lignin, all of which carry negative charges and can effectively adsorb excess Cu [97]. This reduces their penetration into the cytoplasm and enhances Cu tolerance in plants. Under Cu stress, plants exhibit several adaptive responses at the cell wall level. Most notably, there is a marked increase in the lignin content of plants, leading to significant modifications in cell wall structure [95]. As a highly cross-linked biopolymer, lignin forms a particularly robust barrier that effectively limits Cu entry into the cell interior. Furthermore, under Cu stress, plants activate specific modifications in the cell wall biosynthesis pathway, resulting in the increased production of Cu-binding components such as specialized polysaccharides and proteins [98]. It has been observed that when plants experience Cu-induced cell wall damage, they engage in the synthesis of new cell wall components. This process serves to activate repair mechanisms and restore the integrity and functionality of the cell wall. A multitude of enzymes are implicated in the biosynthesis of plant cell walls. For instance, xyloglucan endotransglycosylase/hydrolase (XTH) plays a crucial role in cell wall relaxation during plant cell expansion, which is responsible for the flexibility of plant cell walls [99]. Pectin methylesterases (PMEs) catalyze the hydrolysis of methyl esters in pectin, reducing its degree of methylation and thereby affecting the cell wall’s physical properties [100]. Polygalacturonase (PGL) plays a vital role in regulating the structure of cell walls in plants [101]. Wall-associated kinases (WAKs) are a class of receptor kinases that are closely related to plant cell walls. These proteins play a pivotal role in signal transduction between the cell wall and the cytoplasm, which are essential for cell expansion under Cu stress. The isolation of Cu in vacuoles is achieved via an active transport system on the vacuolar membrane [96], which relies on electrochemical gradients. The activation of transporters such as ATPase (P_1B_-ATPase) and ABC proteins is facilitated by transmembrane pH gradients. The increase in the intracellular Cu concentration prompts the activation of the vacuolar membrane Cu transporter, which facilitates an accumulation of excess Cu in vacuoles, which bind with proteins, organic acids and sugars. This binding process effectively sequesters the Cu ions, thereby mitigating their potential toxicity [29]. When the Cu supply is insufficient, the stored Cu in the vacuoles can be released again through membrane transporters to meet the plants’ growth requirements. In tomato, the addition of nitric oxide (NO) has been shown to promote the isolation of excess soluble Cu in vacuoles by transferring Cu from the cytoplasm to the vacuoles. This process significantly alleviates the toxic effects of Cu [102]. In the presence of excess Cu, the ectopic overexpression of the vacuolar proton pump *TaVP1* in tobacco results in higher catalase activity and the accumulation of more Cu in the roots, when compared to wild-type plants [103]. Collectively, cell wall immobilization and vacuolar sequestration constitute a robust, two-tiered detoxification network that efficiently lowers cytosolic Cu to sub-toxic thresholds, endowing plants with marked Cu tolerance. Nevertheless, the complete inventory of cell wall-modifying enzymes and vacuolar sequestration machinery remains largely unclear, representing a fertile frontier for future discovery.

### 6.3. Metal Efflux

Plants achieve the process of the detoxification through achieving a reduction in metal ion enrichment levels within their tissues, thereby facilitating metal ion efflux. Many studies have identified the critical functions of transporters such as HMA, ZIP, and MTP (metal tolerance protein) in this process. In a study of A. thaliana, it was observed that the knockout of *AtHMA5* resulted in increased Cu accumulation in the roots compared to wild-type plants under excess Cu conditions [29]. The plasma-membrane-localized OsHMA9 enhances plant tolerance to high Cu by actively effluxing excess Cu out of the cell, thereby lowering intracellular Cu levels [104]. The knockout of *OsZIP1* in *O. sativa* was shown to result in a substantial inhibition of plant growth under high Cu stress, while its overexpression promoted growth. The localization of OsZIP1 in the endoplasmic reticulum and the cytoplasmic membrane suggests the possibility of its function as a metal efflux pump under excess-Cu conditions, thereby indicating a regulatory role in environmental changes [105]. MTPs, which function as efflux pumps, are also involved in the efflux or intracellular sequestration of Cu. In recent years, relevant reports have revealed the expression patterns of *MTP* in *Fagopyrum tataricum* under Cu stress. Most *FtMTP* genes can be induced by Cu stress [106]. In *Citrus sinensis*, 12 *CitMTP* genes have been observed to be upregulated under Cu excess [107]. Although there is a paucity of direct reports concerning the function of MTP proteins in the transportation of Cu, their roles in plant metal ion transport and homeostasis are well established. These proteins may play a role in the absorption, distribution and detoxification of Cu, thereby helping plants to maintain a balance of metal ions within their bodies and to mitigate the effects of Cu stress.

### 6.4. Antioxidant Enzymes

To mitigate the negative effects of excess Cu, plants can enhance the activity of antioxidant enzymes, including superoxide dismutase (SOD), peroxidase (POD), catalase (CAT), ascorbate peroxidase (APX), and glutathione reductase (GR). These enzymes play a crucial role in the elimination of ROS and the prevention of oxidative damage. Numerous studies have shown that high Cu concentrations induce the activities of CAT, POD, and SOD [107]. POD is involved in the polymerization of lignin, which enhances the lignification of cell walls. This defense mechanism against heavy metal toxicity increases cell wall rigidity and prevents metal uptake [108]. SOD provides the primary defense against ROS by scavenging superoxide free radicals [109]. APX has been shown to remove H_2_O_2_ in chloroplasts via ascorbic acid as an electron donor [110]. POD and APX in plant cell walls have been shown to be activated under Cu stress; they eliminate Cu-induced ROS and protect cell walls from oxidative damage. In *Camellia sinensis*, an increased concentration of Cu in the leaves has been observed to result in the generation of ROS, thereby inducing an oxidative stress response, accompanied by a notable augmentation in the activities of CAT and POD in the leaves [111]. However, it has also been demonstrated to induce the expression of Cu/Zn-SOD genes in roots and stems, thereby mitigating oxidative stress-induced damage [112]. It is imperative that future research endeavors prioritize the investigation of the gene function of these ROS-related genes in a variety of plant species.

## 7. Transcription Factors in Response to Cu Stress in Plants

Transcription factors are crucial components in signal perception and signal transduction pathways [113]. The WRKY (WRKYGQK domain) [114], ERF (ethylene-responsive transcription factor) [115], NAC, bHLH (basic helix–loop–helix), and MYB (myeloblastosis protein) [116] play indispensable roles in maintaining Cu homeostasis regulation in various plants. For instance, in *A. thaliana*, AtSPL7 (SBP-like Protein 7) is localized in the nucleus and endoplasmic reticulum, as the core transcriptional regulator of Cu deficiency response. It is activated by an imbalance in Cu homeostasis, binding to the *GTAC* motifs in the promoters of *AtFRO4/5* and *AtCOPT1/2/6*, thereby activating their expression and enhancing Cu uptake, which consequently regulate Cu absorption, transport and allocation to adapt to Cu homeostasis imbalance [117,118,119,120,121,122]. In addition, two bHLH family members, CITF1 and CITF2 (Cu-DEFICIENCY INDUCED TRANSCRIPTION FACTOR), are released through interactions with AtSPL7 and subsequently activate the expression of root Cu uptake genes *AtFRO4/5* and *AtCOPT2* [3,15,120,123]. Furthermore, CITF1 and CITF2, and KIN17 and HY5 (ELONGATED HYPOCOTYL 5) can also interact with AtSPL7 to participate in regulating Cu-responsive target genes [124,125]. Moreover, *FIT* (*FER-LIKE IRON DEFICIENCY-INDUCED TRANSCRIPTION FACTOR*) and bHLH family members (*bHLH38*, *bHLH39*, *bHLH100*, and *bHLH101*) can also directly bind to the promoters of Cu uptake genes to activate *AtCOPT2*, *AtFRO4*, and *AtFRO5* for increasing Cu absorption in *A. thaliana* [120,126]. *AtTCP16* (named after TEOSINTE BRANCHED 1, CYCLOIDEA and PROLIFERATING CELL FACTOR 1) can bind to the promoter of *AtCOPT3* to downregulate its expression, thereby altering Cu accumulation and affecting pollen development in *A. thaliana* [127]. Meanwhile, *AtNAC02* may regulate key genes involved in Cu detoxification (such as *COX11* and *HCC1*) to coordinate Cu compartmentalization in vacuoles and mitochondria, thereby reducing Cu toxicity [128].

In *O. sativa*, the *OsWRKY*, *OsbHLH*, and *OsMYB* families have been reported to participate in Cu homeostasis regulation. Through sequence similarity comparison, *OsSPL9* was found to be the homologous gene of *AtSPL7*, which regulated Cu homeostasis under Cu deficiency by controlling Cu uptake and transport genes including *OsCOPT1*, *OsCOPT5*, and *OsYSL16* [8,128,129,130,131]. In addition, *OsMYB84* transcriptionally activates the expression of *OsCOPT2* and *OsHMA5*, which enhances Cu uptake in roots, and promotes Cu translocation to shoots [56]. The knockout of *OsMYB67* downregulates the expression of *OsHMA9*, while it upregulates the expression of *OsATX1* and *OsHMA5*, which promotes Cu allocation to shoots, and increases Cu accumulation in grains [132]. Furthermore, *OsWRKY72* functions as a suppressor of *OsGLP8-7* and lignin biosynthesis genes, and its overexpression inhibits lignin synthesis and cell wall lignification, thereby impairing the barrier function of the cell wall against Cu, and reducing Cu tolerance [133]. *OsWRKY37* is induced by Cu deficiency, binds to the promoters of *OsCOPT6* and *OsYSL16* to promote their expression, which promotes Cu uptake in roots, and facilitates Cu translocation from roots to shoots and spikelets [120]. *OsWRKY37* and *OsWRKY11* are induced by excess Cu, and their regulatory role in Cu deficiency remains unclear [134]. In our study, we also observed that *OsWRKY42* expression is induced to a marked extent under high-Cu conditions and constructed a co-expression network for this (Figure S2); it may also harbor regulatory proteins involved in Cu tolerance.

In other green plants, transcription factors have also been reported in Cu homeostasis regulation. Overexpression of *OsMYB4* in rapeseed plants can improve the tolerance of rapeseed plants to Cu tolerance [116]. In apple, excess Cu induces *MdWRKY11* to promote the expression of plasma membrane-localized MdHMA5,which increases Cu flux into the apoplastic space, and enhances tolerance to Cu in apple [135]. In *Chlamydomonas reinhardtii*, Cu response regulator1 (*CrCRR1*) activates the *CYC6* gene (encoding Cyt c6) via Cu response elements (*CuREs*) in response to Cu deficiency, which maintain the stability of the photosynthetic electron transport chain [136]. In *P. patens*, both *PpSBP2* and *PpSBP1* transcription factors negatively regulate the *FeSOD* gene, thereby participating in the regulation of ROS homeostasis [137]. *CrCRR1*, *PpSBP2*, and *PpSBP1* all contain a plant-specific *SBP* domain involved in Cu response. In *T. aestivum*, the expression level of *TaWRKY74* is significantly induced by 50 μM Cu, which positively regulates the transcription of Glutathione S-transferase (*TaGST1*), thereby regulating GSH content to cope with Cu stress [138]. In soybean (*Glycine max*), members of the *Golden2-Like* (*G2-Like* or *GLK*) transcription factors have been reported to respond to Cu stress, among which *GmGLK1*, *GmGLK5*, *GmGLK13*, *GmGLK67* and *GmGLK129* are significantly induced under Cu stress, but *GmGLK74* and *GmGLK106* show a dynamic trend of downregulation first, and then upregulation, followed by downregulation under 6 h of Cu stress [139].

## 8. Conclusions and Future Perspectives

Cu is indispensable for plant growth and development, and a deficiency or excess of it severely impacts crops productivity. Our review highlights the sophisticated regulatory networks governing Cu homeostasis in plants, encompassing uptake, transport, distribution, and detoxification. Specifically, the COPT family mediates high-affinity Cu uptake, with OsCOPT1, OsCOPT5, and OsCOPT7 playing pivotal roles in root absorption and shoot translocation [27]. Root exudates (citrate and proline) chelate Cu, which might be reduce bioavailability. Cell wall binding and vacuolar compartmentalization limit cytoplasmic Cu accumulation [56,132]. Antioxidant enzymes (SOD, CAT, and APX) counteract Cu-induced oxidative stress. Tissue-specific expression profiles reveal that *COPT* genes are highly active in roots and vascular tissues (Figure 3). There is an evolutionary divergence of COPT families in monocots and eudicots, suggesting species-specific adaptations to Cu stress (Figure 2).

Future research should investigate how transcription factors (e.g., *OsMYB84*) coordinate *COPT* expression under varying Cu levels of stress. The phosphorylation and ubiquitination of transporters (e.g., OsCOPT2) in response to Cu stress should be explored. In addition, key vacuolar sequestration genes (e.g., *OsCOPT7*) could influence Cu translocation to grains, which may hold significant potential for addressing human dietary Cu deficiency. Furthermore, crops with hyperaccumulation traits (e.g., enhanced *OsCOPT* expression) might be developed for use on Cu-polluted farmland. These findings might also improve human health. For instance, modulating the Cu distribution of grains via *OsCOPT* could be useful in combating Cu deficiency in human diets. Although genetic engineering techniques can efficiently address the practical issue of Cu imbalance, their application in agricultural production still poses certain risks, including disrupting ecological balance. Therefore, it is crucial to conduct thorough environmental risk assessments and controlled field trials before applying such genetically modified crops in agriculture. In addition, it also important to investigate the interplay between Cu homeostasis and other stresses (e.g., salinity, pathogens), and study how drought/flooding alters Cu solubility in soils and its uptake by crops. As Cu pollution escalates, integrating molecular biology, agronomy, and environmental science will be key to sustaining crop production. By deciphering Cu homeostasis mechanisms and deploying biotechnological tools, we might cultivate resilient crop varieties for protecting ecosystems and ensuring global food supplies.

## Supplementary Materials

The following supporting information can be downloaded at https://www.mdpi.com/article/10.3390/plants14172710/s1: Figure S1. Expression analysis of *COPTs* in rice. Data were downloaded from a public database (https://rice-singlecell.shinyapps.io/orvex_app/ [accessed on 10 July 2025)]). Figure S2. Gene co-expression network of transcription factor *OsWRKY42*. Genes from profiles *OsWRKY42* were analyzed and identified using a gene co-expression network with the k-core algorithm. Cycle nodes represent genes, the size of nodes represents the power of the interrelation among the nodes, and edges between two nodes represent interactions between genes. The more edges on a gene, the more genes connecting to it, and the more central role it has within the network.
