# Supplementary material for: Molecular Evolution of Cu Transporters and Transcription Factors in Plant Response to Copper Stress
Source: Plants (Basel). 2025 Sep 1;14(17):2710. doi: 10.3390/plants14172710 (PMC12430517; doi:10.3390/plants14172710)
Supplement: Supplementary file 1 [file plants-14-02710-s001.zip › plants-3810293-supplementary.pptx]

## Slide 1
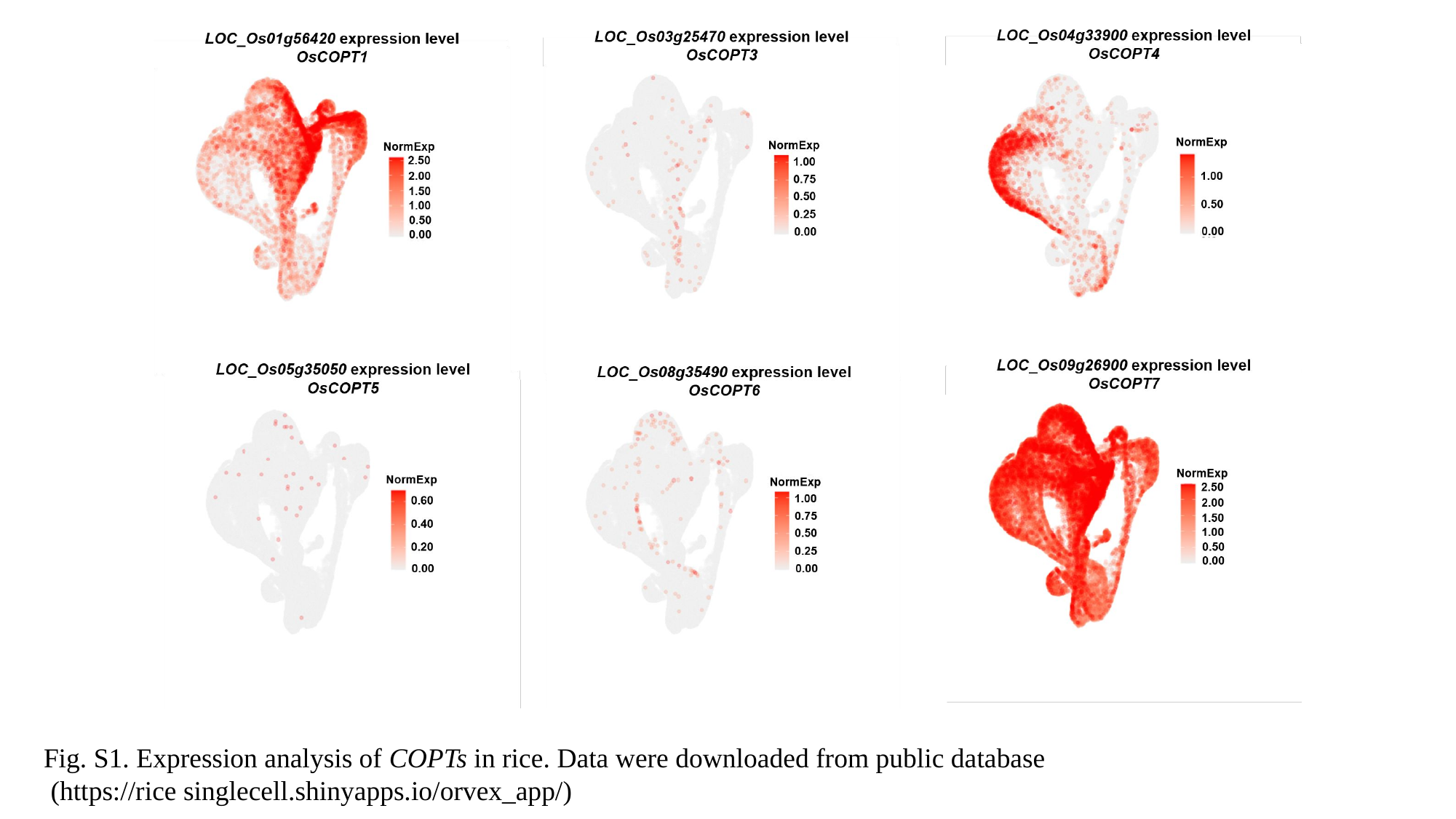

Fig. S1. Expression analysis of COPTs in rice. Data were downloaded from public database
 (https://rice singlecell.shinyapps.io/orvex_app/)

## Slide 2
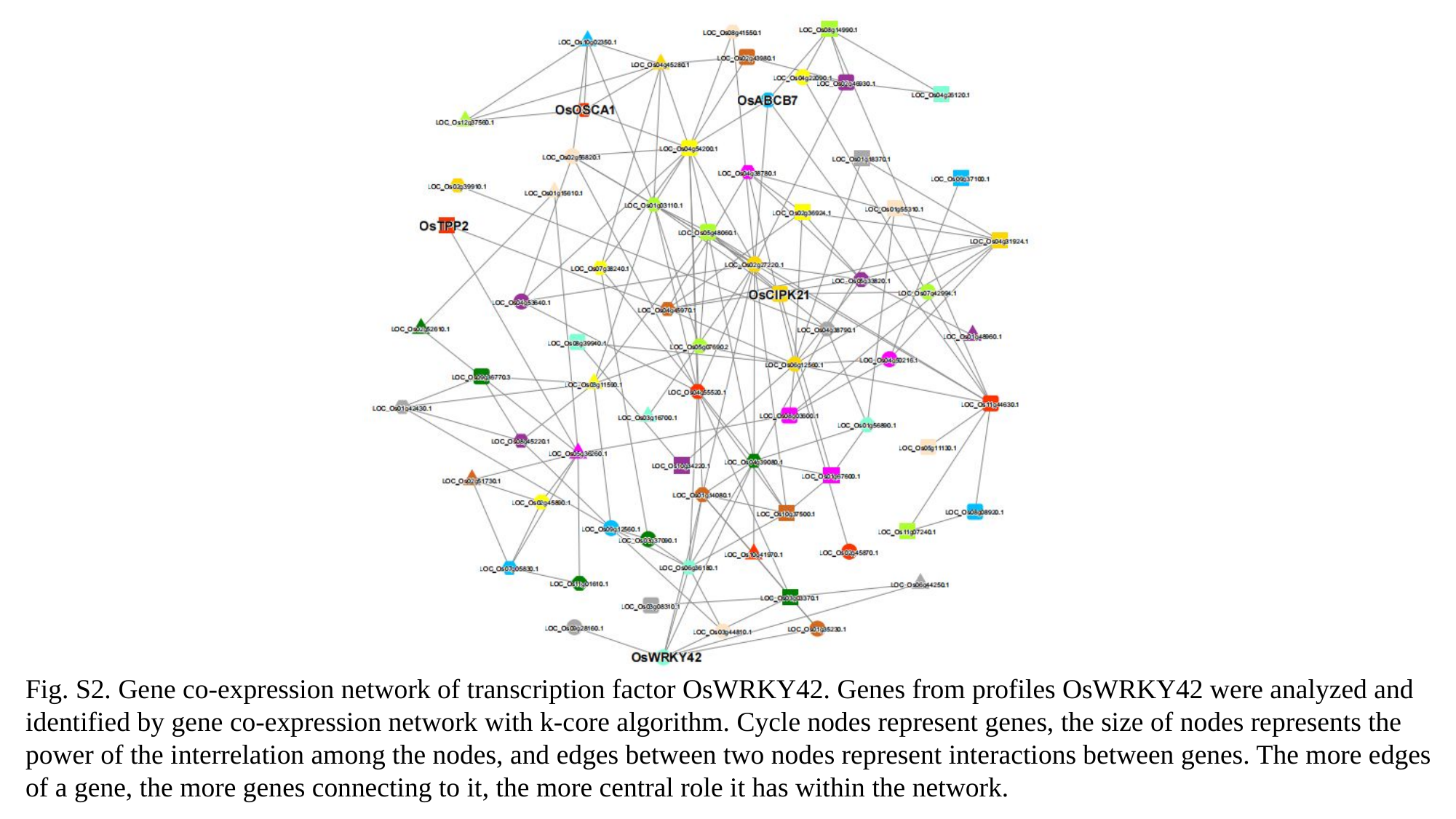

Fig. S2. Gene co-expression network of transcription factor OsWRKY42. Genes from profiles OsWRKY42 were analyzed and identified by gene co-expression network with k-core algorithm. Cycle nodes represent genes, the size of nodes represents the power of the interrelation among the nodes, and edges between two nodes represent interactions between genes. The more edges of a gene, the more genes connecting to it, the more central role it has within the network.
